# Axillary vein thrombosis related to midline catheter in a patient with Wilson’s disease: a case report

**DOI:** 10.3389/fmed.2026.1752624

**Published:** 2026-04-15

**Authors:** Lei He, Huan Zhou, Pengfei Xu, Xiupei Xu, Ping Wang, Hui Han

**Affiliations:** 1 First Clinical Medical College, Anhui University of Chinese Medicine, Hefei, China; 2 Department of Neurology, The First Affiliated Hospital of Anhui University of Chinese Medicine, Hefei, China; 3 Department of Geriatric Cardiovascular Medicine, The First Affiliated Hospital of Anhui University of Chinese Medicine, Hefei, China

**Keywords:** liver cirrhosis, midline catheter, splenectomy, upper extremity deep vein thrombosis, Wilson’s disease

## Abstract

Wilson’s disease (WD) is a rare hereditary disorder of copper metabolism that requires long-term copper-chelating therapy. In China, cyclic intravenous sodium dimercaptopropanesulfonate (DMPS) is used in hospitalized patients to enhance copper excretion, whereas oral chelating agents are typically used for maintenance therapy. We report a 44-year-old woman with WD complicated by liver cirrhosis and prior splenectomy who developed left axillary vein thrombosis six days after midline catheter placement for intravenous DMPS infusion. Baseline evaluation showed WD-related hepatic and neurological involvement, with normal conventional coagulation parameters but evidence of platelet activation. Duplex ultrasound confirmed axillary vein thrombosis. The patient was treated with oral rivaroxaban for 3 months, resulting in complete symptom resolution. Follow-up ultrasound suggested recanalization of the affected vein, and no recurrence of thrombotic events occurred during 6 months of follow-up. This case highlights a potential risk of catheter-related thrombosis in WD patients with additional prothrombotic factors and underscores the importance of careful venous access management during inpatient intravenous therapy.

## 1 Introduction

Wilson’s disease (WD) is an autosomal recessive disorder of copper metabolism with a global prevalence of 1/40,000–1/50,000 and is characterized by progressive liver dysfunction and central nervous system degeneration (1, 2). In most international guidelines, long-term management of WD is based on oral chelating agents or zinc therapy (3, 4). However, in certain regions, including China, sodium dimercaptopropanesulfonate (DMPS) is used in hospitalized patients as part of cyclic anti-copper therapy, particularly in selected clinical settings requiring intensified copper removal (5–8).

Because cyclic intravenous therapy requires repeated venous access, peripherally inserted midline catheters (MCs) are commonly used for medium-term infusion management (9). However, WD patients often have comorbidities such as liver cirrhosis and post-splenectomy status, which may be associated with an increased thrombotic risk. These conditions may alter vascular endothelial function and coagulation homeostasis, and such disease-specific factors may not be fully captured by conventional thrombosis risk assessments. As a form of peripheral intravenous access, MCs are subject to intravenous line-associated complications, including catheter-related thrombosis, local infection, phlebitis, occlusion, and mechanical complications such as malposition or dysfunction. While these complications are well recognized in general infusion practice, evidence specifically describing catheter-related complications in patients with WD receiving cyclic intravenous chelation remains limited. In this context, we present a case of a WD patient with liver cirrhosis and post-splenectomy status who developed MC-related axillary vein thrombosis. This case highlights a potential risk of catheter-related thrombosis in this clinical setting and underscores the need for increased awareness and further research on venous access management in patients with WD.

## 2 Case description

A 44-year-old female patient with a known diagnosis of WD was admitted to The First Affiliated Hospital of Anhui University of Chinese Medicine on August 22, 2024, for her sixth cycle of DMPS-based copper-chelating therapy. The patient had a history of WD complicated by liver cirrhosis and had undergone splenectomy 5 years earlier for hypersplenism secondary to cirrhosis. On admission, she had no overt neurological symptoms such as tremor, dysarthria, or gait disturbance, reported bilateral knee pain, had a history of Kayser–Fleischer rings documented based on prior ophthalmologic examination (although no recent slit-lamp evaluation was available), and no renal abnormalities were identified.

DMPS was administered as cyclic inpatient therapy in this case. The treatment plan consisted of three cycles of DMPS (0.5 g/day diluted in 250 mL of 0.9% sodium chloride for 6 consecutive days), followed by a 2-day interval with oral zinc gluconate and intravenous calcium gluconate, for a total planned course of 24 days. The number of cycles was determined based on the patient’s clinical condition. This regimen required hospitalization due to repeated infusions, clinical monitoring, and venous access management.

**Figure 1:**
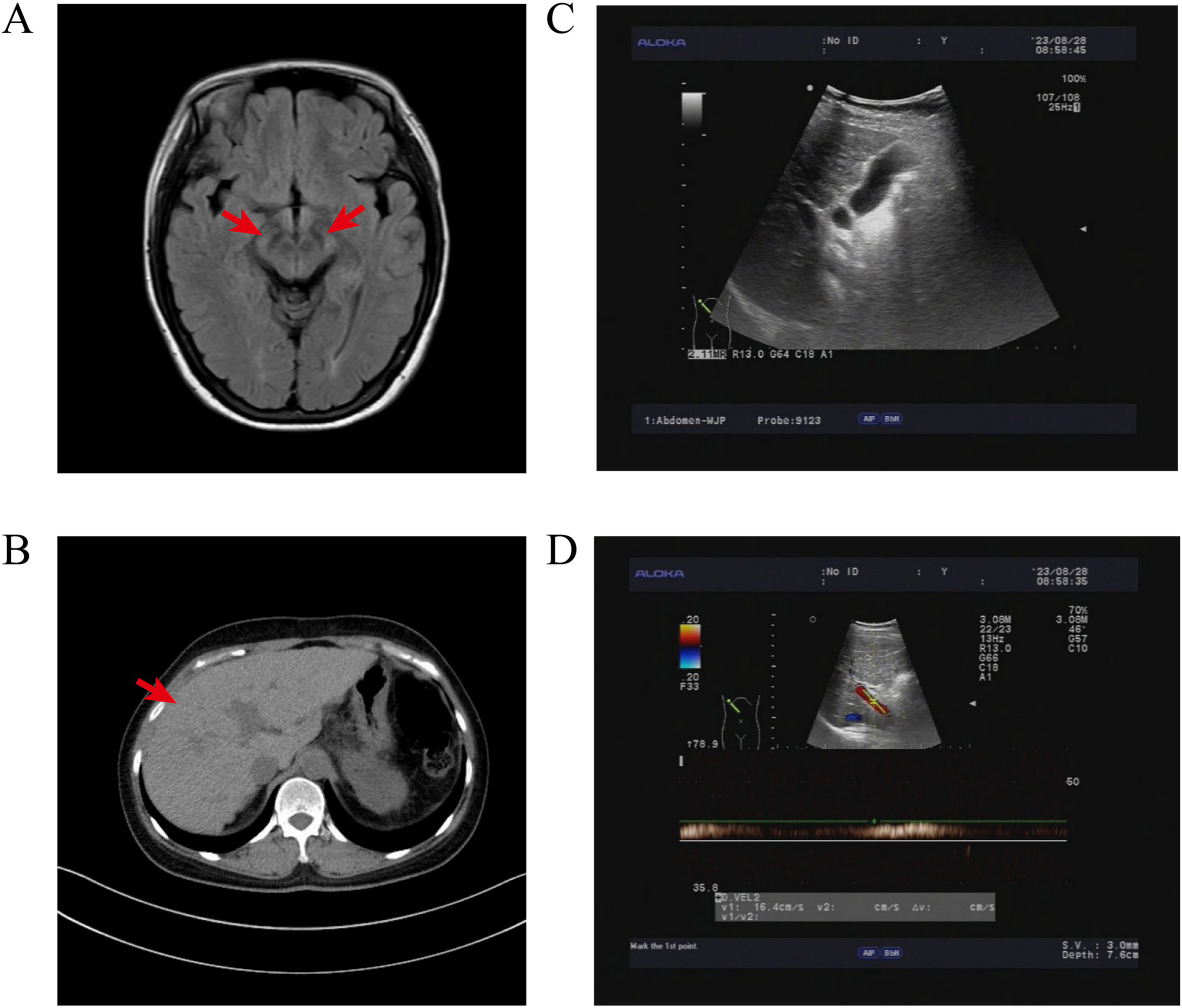
Imaging findings of the patient with WD. **(A)** Cranial MRI T1WI showed symmetrical abnormal signals in the bilateral cerebral peduncles, consistent with the typical manifestation of central nervous system involvement in WD; **(B)** chest CT revealed an irregular liver contour, uneven margin, and heterogeneous hepatic parenchymal density; no splenic structure was observed in the splenic region, suggesting post-splenectomy status; **(C)** liver color Doppler ultrasound demonstrated coarse and enhanced intrahepatic echoes with uneven distribution, suggesting cirrhotic changes in the liver; **(D)** the portal vein blood flow spectrum was normal (peak velocity [Vmax] = 16.4 cm/s).

Baseline imaging demonstrated symmetrical abnormalities in the bilateral basal ganglia on cranial MRI, as well as features of liver cirrhosis and post-splenectomy changes on chest CT and abdominal ultrasound (Figure 1). Laboratory tests showed abnormalities consistent with WD, elevated liver fibrosis markers, normal coagulation indices, and evidence of platelet activation (Table 1); the ALT level was 29.8 U/L, and CRP was not measured.

**Table 1:** Baseline examination results of the patient on admission.

Category	Specific indicator	Test value (Reference Range)
Core diagnostic	Ceruloplasmin	0.075 g/L (0.23–0.44 g/L)
Indicators for WD	Ceruloplasmin oxidase	0.05 U (0.26–0.65 U)
	Serum copper	2.83 μmol/L (11–24.4 μmol/L)
	24-h urinary copper	1007.36 μg/24 h
	Serum zinc	12.2 μmol/L (9.2–22.9 μmol/L)
Liver fibrosis-related	GGT	68 U/L (7-45 U/L)
Indicators	Fibronectin	401.3 mg/L (250-400 mg/L)
	Hyaluronic acid	133.27 ng/mL (<120 ng/mL)
Coagulation and thrombosis-related	PT	10.6 s (9–13 s)
	INR	0.97 (0.89–1.25)
Indicators	APTT	32.2 s (25–33.8 s)
	Fibrinogen	2.64 g/L (2-4 g/L)
	TT	20.6 s (14–21 s)
	Platelet count	208 × 10^9^/L (125–350 × 10^9^/L)
	Large platelet ratio	50% (13–38%)
	Reticulocyte percentage	1.57% (0.5–1.5%)

GGT, γ-glutamyl transferase; PT, prothrombin time; INR, international normalized ratio; APTT, activated partial thromboplastin time; TT, thrombin time.

Due to the prolonged therapy course requiring repeated infusions, a peripherally inserted MC was placed to facilitate venous access. On hospital day 3, a 4F single-lumen MC was inserted into the left basilic vein via blind puncture by an experienced nurse (>5 years of venous access experience). The procedure was completed without immediate complications, and catheter patency was confirmed by unobstructed blood return. After catheter placement, routine catheter care and clinical monitoring were performed: the insertion site and upper extremity were assessed daily for redness, swelling, pain, induration, and changes in arm circumference; laboratory monitoring included coagulation parameters and platelet-related indices; duplex ultrasound was performed when catheter-related thrombosis was clinically suspected and was used for follow-up assessment.

**Figure 2:**
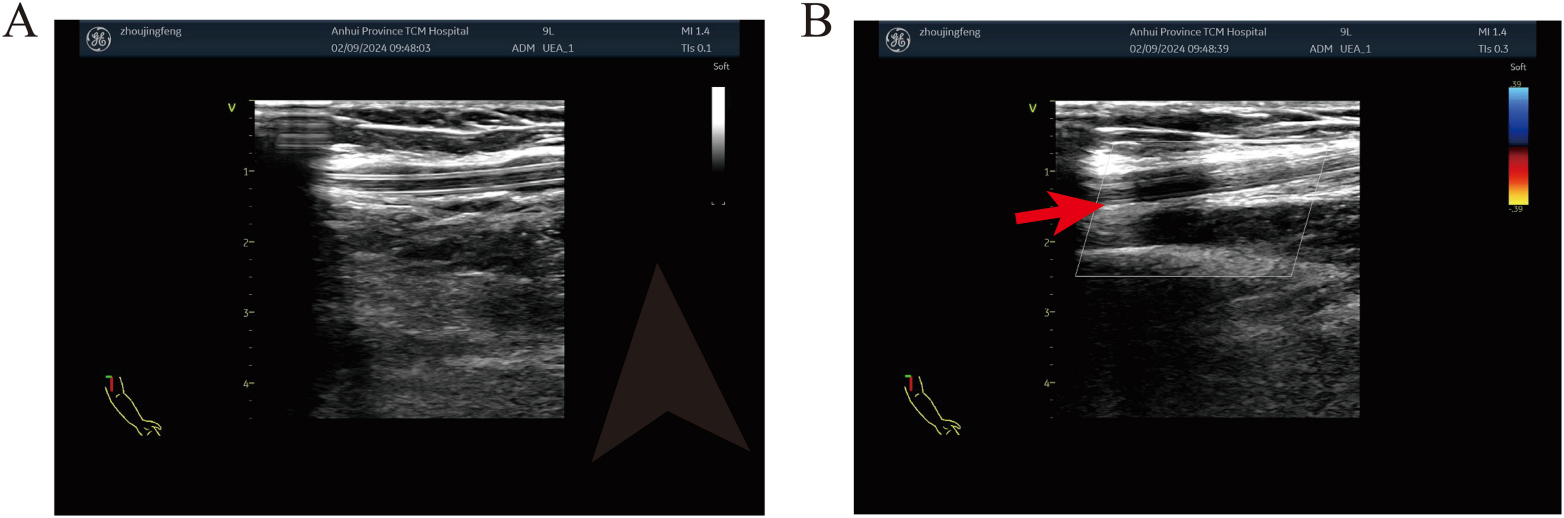
Duplex ultrasound findings of the left upper extremity. **(A)** Grayscale ultrasound image showing the axillary vein. **(B)** Color Doppler ultrasound demonstrating reduced or absent flow in the affected vein (arrow), consistent with thrombosis.

On day 3 after MC placement, the patient developed mild redness and swelling above the left elbow, which did not improve with local hot compresses. These findings were suggestive of superficial thrombophlebitis; however, no ultrasound examination was performed at that stage. By day 6, symptoms worsened, with increased pain and an increase in left upper arm circumference from 25 cm to 26 cm, and duplex ultrasound confirmed left axillary vein thrombosis (Figure 2).

Following the diagnosis of thrombosis, the patient was evaluated by the vascular surgery team, and oral rivaroxaban (15 mg/day) was initiated for anticoagulation. The MC was initially retained because the patient still required ongoing DMPS infusion and had limited peripheral venous access for re-puncture, and after one week of treatment, local symptoms resolved, arm circumference returned to baseline, and no clinical signs suggestive of pulmonary embolism (e.g., chest pain or dyspnea) were observed. The patient subsequently requested discharge before completion of the third DMPS cycle, and the MC was removed prior to discharge; she continued outpatient anticoagulation with rivaroxaban (10 mg/day) for 3 months. During follow-up, symptoms resolved completely and no thrombotic recurrence was reported, duplex ultrasound performed at a local hospital was reported as normal, suggesting recanalization of the affected vein, although the original report was not available for independent review, and no clinical signs suggestive of infective endocarditis were observed during hospitalization, with no further diagnostic evaluation performed.

## 3 Discussion

MCs have become a common venous access option for patients requiring medium-term intravenous therapy (1–4 weeks), as they combine the ease of peripheral access with the advantages of longer indwelling duration. Although guidelines recognize their value in selected clinical scenarios, catheter-related thrombosis remains an important complication, with reported incidence ranging from 0.7 to 11.88% across populations, and relevant risk factors still require further clarification (10–14).

Currently, studies on MC-related thrombosis focus primarily on pediatrics, critical care, and geriatrics, while evidence in patients with WD remains limited. In addition, thrombotic events have been reported in patients with WD, suggesting that coagulation imbalance may contribute to thrombotic risk in certain clinical contexts (15). This case highlights a rare but clinically relevant vascular complication in a WD patient with cirrhosis and post-splenectomy status. Below, we discuss possible contributing factors, management considerations, and practical implications based on this case and the available literature, with particular emphasis on the importance of oral chelation in reducing WD-related iatrogenic complications.

### 3.1 Factors potentially contributing to thrombosis

WD is characterized by abnormal copper metabolism, and chronic hepatic involvement may further affect vascular endothelial function and coagulation balance (16–20). In this patient, decreased ceruloplasmin and serum copper levels were consistent with WD, while elevated liver fibrosis markers suggested chronic hepatic injury. However, in WD, total serum copper is largely influenced by ceruloplasmin levels and does not reliably reflect total body copper burden. In this case, the markedly elevated 24-h urinary copper (1007.36 μg/24 h) indicated ongoing copper overload, thereby supporting the need for continued intravenous chelation therapy despite the low serum copper level. The patient also had a history of splenectomy for WD-related hypersplenism. Although her platelet count was normal on admission, the large platelet ratio (50%) was markedly higher than the normal range (13–38%), which may reflect increased platelet activation and a potentially prothrombotic background (21, 22). In addition, the patient was a 44-year-old perimenopausal woman, and hormonal fluctuations may have had an additional influence on thrombosis risk.

As the core drug for copper-chelating therapy in this case, DMPS has no clear reports of MC-related thrombosis risk. More broadly, the safety of administering potentially irritant drugs through MCs remains controversial. Some studies suggest that drugs with extreme pH values—such as vancomycin, acyclovir, and doxycycline—may increase the risk of MC-related thrombosis (23), whereas other studies have not found an increased risk with short-term infusion of such agents (24, 25). In this context, infusion-related factors such as drug properties and local vascular tolerance may influence catheter-related complications. In the present case, DMPS infusion may have interacted with the patient’s underlying vascular vulnerability, although its specific contribution to thrombosis remains uncertain.

Catheter-related mechanical and hemodynamic factors were also likely relevant in this case. Existing studies have shown that the thrombosis rate of 4F MCs is lower than that of 5F MCs, with no significant difference compared with 3F MCs (26). In this case, a 4F MC was used; however, thrombosis still occurred. It is possible that the patient’s underlying cirrhosis and altered vascular condition reduced tolerance to catheter-related mechanical stress.

Furthermore, catheter tip position may influence thrombosis risk (27). Previous studies have suggested that when the MC tip is located near the axillary-subclavian vein junction, endothelial irritation may be greater and thrombosis risk may be higher (28, 29). In this case, thrombosis occurred in the left axillary vein. Because the catheter was inserted by blind puncture, the exact tip position could not be confirmed. It is therefore possible that local endothelial irritation and altered blood flow contributed to thrombosis development.

Taken together, these findings suggest that catheter-related mechanical and hemodynamic factors likely acted as the immediate trigger for thrombosis, while WD-related metabolic abnormalities, cirrhosis, and post-splenectomy status may have created a prothrombotic background that increased susceptibility.

### 3.2 Management considerations

Management of MC-related thrombosis requires balancing the need for catheter retention against the risk of complications. Previous reports suggest that when the catheter remains functional and ongoing treatment is required, short-term retention under anticoagulant therapy may be considered in selected cases (30–32). In this case, the MC was not immediately removed after the diagnosis of upper-extremity deep vein thrombosis because the patient required ongoing copper-chelating therapy and had limited peripheral venous access. In addition, ultrasound showed that the lumen was not completely occluded and that there were no signs of floating thrombus, indicating no urgent need for immediate catheter removal. After consultation with the vascular surgery department, short-term catheter retention combined with anticoagulant therapy was considered a reasonable individualized approach.

Rivaroxaban was selected because it is an established option for venous thromboembolism and offers the practical advantage of oral administration without routine laboratory monitoring (33–35). In this patient, the dosing regimen was individualized based on the overall clinical context, including cirrhosis and concern about bleeding risk. For the outpatient phase, rivaroxaban was continued at 10 mg/day for 3 months (36, 37). The patient achieved favorable clinical outcomes, with complete resolution of local symptoms and no thrombus recurrence during follow-up.

### 3.3 Clinical implications

This case suggests several practical considerations for MC use in WD patients who require intravenous therapy in specific clinical settings.

First, in WD patients with additional thrombotic risk factors such as cirrhosis or post-splenectomy status, individualized risk assessment may be helpful when selecting vascular access. Pre-procedural ultrasound evaluation of vein characteristics and attention to catheter-to-vein ratio may also help reduce thrombosis risk. Second, ultrasound-guided insertion and attention to catheter positioning may be helpful in reducing mechanical irritation and thrombosis risk. Selection of an appropriately sized catheter may also be important when prolonged intravenous therapy is anticipated. Third, close monitoring of the puncture site and upper extremity during inpatient infusion therapy is important for early detection of complications. When early warning signs such as redness, swelling, pain, or increasing arm circumference occur, prompt ultrasound evaluation may facilitate early diagnosis and treatment.

More broadly, this case also underscores the importance of oral chelation as the standard long-term treatment strategy for most patients with WD, in keeping with current international guidance. Intravenous chelation is typically reserved for specific clinical situations and should not replace maintenance oral therapy. Reducing reliance on repeated hospitalization and intravenous access may help decrease the risk of IV line-related complications.

This report has limitations. A formal thrombophilia panel was not performed in this case, and routine coagulation parameters alone cannot exclude underlying thrombophilia. However, the patient had no prior history of thrombotic events, and the thrombosis developed shortly after midline catheter placement, suggesting a strong temporal association. Therefore, catheter-related thrombosis is considered the most likely explanation in this clinical context, although an underlying predisposition cannot be completely excluded. As a single case, it cannot fully clarify the relative contributions of WD-related factors, DMPS infusion, and catheter-related mechanical factors to thrombosis development. In addition, infective endocarditis was excluded based on clinical assessment rather than formal diagnostic testing. Furthermore, follow-up ultrasound findings were based on an external hospital report that was not available for independent review.

## 4 Conclusion

To our knowledge, this is among the first reported cases of MC-related axillary vein thrombosis in a WD patient with cirrhosis and post-splenectomy status. During 6 months of follow-up, the patient achieved clinical resolution without recurrence under an individualized anticoagulation strategy. This case highlights several practical considerations for venous access management in this specific clinical context. Conventional thrombosis risk assessment tools may not fully capture the risk in WD patients with additional factors such as cirrhosis and prior splenectomy, and individualized catheter management may be important when prolonged intravenous therapy is required. Further studies are needed to better define thrombosis risk and optimal management strategies in this population.

## Data Availability

The original contributions presented in the study are included in the article/supplementary material, further inquiries can be directed to the corresponding author.
